# Determination of the Long-Term Stability of Propofol in Human Plasma Frozen for Eight Years and Quantified by UHPLC–MS/MS

**DOI:** 10.1155/ianc/7533068

**Published:** 2025-08-22

**Authors:** Stefanie Schmidt, Harald Ihmsen, Tino Münster, Jürgen Schüttler, Christian Jeleazcov, Andreas Wehrfritz

**Affiliations:** ^1^Department of Anesthesiology, University Hospital Erlangen, Friedrich-Alexander-Universität Erlangen-Nürnberg (FAU), Erlangen, Germany; ^2^Department of Anesthesia and Intensive Care Medicine, Krankenhaus Barmherzige Brüder Regensburg, Regensburg, Germany

## Abstract

The intravenous anesthetic propofol is frequently used for the induction and maintenance of general anesthesia. Propofol has, however, a potential for abuse, and it has been involved in suicide deaths particularly among medical personnel. The long-term stability of propofol concentrations in human plasma samples when stored under normal laboratory conditions over several years might be important for forensic toxicology. The present study investigated the long-term stability of propofol after storage at −20°C for eight years. For this purpose, 67 plasma samples from five patients, who had undergone a former clinical trial, were re-analyzed. Plasma samples were extracted using protein precipitation. Propofol plasma concentrations were determined by ultra-performance liquid chromatography (UPLC) with gradient elution, followed by tandem mass spectrometry with electrospray ionization. Deuterium-labeled propofol was used as an internal standard. The assay was linear in the range of 50–10,000 ng/mL with a limit of detection of 0.5 ng/mL and a lower limit of quantification of 50 ng/mL, respectively. Accuracy and precision were high with intra- and interassay errors within ±5%. The median relative deviation between the measurements in 2023 and 2015 was −7.0% (interquartile range: −19.7% and 5.3%). Therefore, propofol concentrations in human plasma can be considered relatively stable in samples frozen at −20°C over eight years, and plasma samples stored under these conditions might be used for forensic purposes.

**Trial Registration:** ClinicalTrials.gov identifier: NCT02199067

## 1. Introduction

Propofol is a frequently used intravenous anesthetic agent known for its rapid onset and short duration of action [1]. It is commonly administered during surgeries and diagnostic procedures to induce and sustain anesthesia or sedation. Its popularity in clinical practice is largely due to its favorable pharmacokinetic profile, allowing for a precise control over a wide range of sedation levels [1, 2]. Propofol is not only used for short-term anesthesia in emergency medicine, for medium-term applications in anesthesia but also for long-term sedation in intensive care medicine [3]. Propofol has some advantages over balanced anesthesia, in which anesthetic gases such as sevoflurane are used, and is therefore being administered more and more frequently. It significantly reduces the incidence of postoperative nausea and vomiting (PONV) [4]. In addition, the use of propofol is increasing worldwide as anesthetic gases have been identified as harmful to the environment, as they contribute to global warming [5]. On the other hand, propofol also causes side effects such as euphoria and sexual dreams, which can lead to abuse of the substance [6]. This potential for abuse has been known not only since the death of Michael Jackson in 2009 but has been apparent relatively quickly after the introduction of the drug in the mid-1980s, and the first case reports describing the abuse and addictive potential were published in the 1990s [7]. In a literature research, Uzbay came across a considerable number of 32 case reports dealing with this topic [6]. It is hardly surprising that many of the tragic cases involve people who work in healthcare and even in anesthesiology as doctors or nurses, who have experience with the substance and should be aware of the risks such as respiratory depression or even respiratory arrest when using propofol. This significant number of case reports shows that courts and forensic pathologists will continue to debate the question of propofol concentrations in specimens. It is also possible that stored blood or plasma samples may need to be reanalyzed years later to resolve legal issues. Therefore, the long-term stability of propofol in human plasma when stored over years at normal laboratory conditions is an important issue in forensic toxicology [8]. Bienert et al. found no significant decrease in propofol plasma concentration up to 60 days [9]. Similarly, Li et al. found that the concentrations of propofol remained stable in plasma samples stored at −20°C for up to 14 weeks [10]. There are, however, no data on the stability of propofol concentrations in human plasma for longer periods. In the present study, the long-term stability of 67 human plasma samples was examined using liquid chromatography with tandem mass spectrometry (LC–MS/MS), which is a highly sensitive and accurate technique for analyzing drugs such as propofol in biological matrices [11]. The plasma samples were frozen in 2015 at −20°C and were analyzed again eight years later to determine the decrease in propofol plasma concentrations when stored under these conditions.

## 2. Materials and Methods

### 2.1. Samples

The analyzed samples were collected at the University Hospital Erlangen in 2014 and 2015 in a clinical pilot study on the pharmacokinetics of propofol when measured in blood and in exhaled air. The study was conducted in accordance with the guidelines for good clinical practice and the Declaration of Helsinki. Ethical approval was obtained from the institutional review board (Ethikkommission der Medizinischen Fakultät der Friedrich–Alexander-Universität Erlangen–Nürnberg, Erlangen, Germany; date of approval: 12.02.2014; reference no. 13_14B). Written informed consent was obtained from the participants before enrollment. For induction and maintenance of anesthesia, patients undergoing abdominal surgery received propofol (propofol 2% medium-chain triglyceride [MCT] Fresenius, Fresenius Kabi, Bad Homburg, Germany) as target-controlled infusion (TCI) with plasma target concentrations of 2–5 μg/mL. During infusion and up to 120 min after the end of infusion, arterial blood samples of 8 mL each were drawn into plastic tubes containing ethylenediaminetetraacetic acid (S-Monovette Kalium EDTA, Sarstedt, Nürnbrecht, Germany). The samples were kept at 4°C on ice water, and the plasma was separated by centrifugation within 15 min and was stored at −20°C until analysis, which was performed within two months after sampling. In 2015, the propofol plasma concentrations were determined by a sensitive and specific LC–MS/MS as described by Eisenried et al. [12]. Plasma samples from this study were stored at −20°C. For the present study, 67 available plasma samples from five patients were thawed in 2023, centrifuged at 5000 rpm (2370 × g) at 4°C, and re-quantified, using an adapted and optimized method, which was scaled up from high-performance LC (HPLC) to ultra-HPLC (UHPLC).

### 2.2. Drugs and Reagents

Pure standard solutions of propofol and deuterium-labeled propofol-d17 were purchased from LGC Standards (Wesel, Germany). The following solutions from Sigma-Aldrich (Steinheim, Germany) were used: formic acid, ammonium formate, methanol, acetonitrile, water LC–MS grade, triethylamine, and 2-fluoro-1-methylpyridinium-p-toluenesulfonate (FluMP). Drug-free human serum from Bio-Rad (Feldkirchen, Germany) was used to dilute the standard stock solutions.

### 2.3. Standard Solutions

The following solutions were prepared by diluting a standard stock solution at a concentration of 1 mg/mL in methanol. Quality control (QC), validation, and calibration curve samples were then prepared by adding 50 μL of the diluted standard solution in methanol to 450 μL of drug-free plasma. Calibration curves were prepared with concentrations ranging from 50 to 10,000 ng/mL. The internal standard propofol-d17 was prepared at a concentration of 1000 ng/mL in methanol. QC samples were prepared at concentrations of 100, 1000, and 7500 ng/mL.

### 2.4. Sample Pretreatment

For purification, protein precipitation was employed. Specifically, 200 μL of methanol containing propofol-d17 at a concentration of 1000 ng/mL was added to 50 μL of the plasma sample. For zero samples and blank samples, 50 μL of drug-free plasma was used. The mixture was vortexed and centrifuged for 10 min at 4°C at 5000 rpm (2370 × g). To increase sensitivity, the extracts underwent derivatization as described by Thieme et al. [13]. For this purpose, 40 μL of the supernatant was combined with 80 μL of freshly prepared FluMP solution (25 mg/mL FluMP in acetonitrile) and 20 μL of triethylamine. The mixture was vortexed, centrifuged for 1 min, and then left to rest for 10 min at room temperature. The derivatized samples were then dried under a gentle nitrogen stream. To reconstitute the samples, 200 μL of LC–MS grade water containing 0.1% formic acid was added, followed by vortex mixing. The mixture was centrifuged again for 1 min, and the final solution was transferred to an HPLC vial for analysis.

### 2.5. Equipment

Unlike the method used in 2015, this study utilized an ACQUITY ultra-performance LC (UPLC) H-Class system from Waters (Eschborn, Germany), with adjustments made to optimize the method for this equipment. For the analytical column and separation, a Kinetex Biphenyl column (Phenomenex, Aschaffenburg, Germany) was used, featuring a 1.7 μm particle size and dimensions of 50 × 2.1 mm, in contrast to the previously used C18 column. The UPLC system was coupled with an Xevo TQD mass spectrometer from Waters, equipped with an ESI source. Data acquisition and analysis were performed using MassLynx software, Version 4.2.

### 2.6. UHPLC Conditions

Chromatographic separation was performed using gradient elution with the following mobile phases: Phase A consisted of water with 0.1% formic acid and 5 mM ammonium formate, while Phase B was acetonitrile containing 0.1% formic acid. The flow rate was set at 0.4 mL/min, and 5 μL of the final extraction sample was injected into the system. The gradient started at 90% Phase A, decreasing to 20% within 1 min. This composition was maintained for 1.5 min before returning to the initial conditions. The total run time was 5 min, with the column temperature maintained at 30°C.

### 2.7. MS/MS Conditions

Tandem MS analysis was conducted using positive electrospray mode with multiple reaction monitoring (MRM). The MS parameters were configured as follows: the ion source temperature was set to 120°C, with a capillary voltage of 3.5 kV and a cone voltage of 60 V. The cone gas flow rate was maintained at 50 L/min, while the desolvation gas temperature was kept at 450°C, with a flow rate of 900 L/min. Nitrogen was used as the desolvation cone gas, and argon served as the collision gas. For quantification, the following product ions were monitored: 270.1 ⟶ 133 m/z for the propofol derivative, and 287.1 ⟶ 146.1 m/z for the propofol-d17 derivative. The dwell time for all ion transitions was set to 163 ms.

### 2.8. Quantification and Weighing Factor

For the quantification of the propofol derivative response, the following formula was used:

(1) $$\begin{array}{c} \text{response}=\frac{{\text{Area}}_{\text{propofol⁣derivative}}}{{\text{Area}}_{\text{propofol}-d17\text{ derivative}}}\cdot {\text{Conc}}_{\text{propofol}-d17}. \end{array}$$

This formula quantifies the response of the analyte in relation to the internal standard, ensuring accurate and consistent measurement of the analyte concentration. The propofol plasma concentration was obtained from the response by applying the calibration curve. To account for the derivatization with FluMP, the concentrations determined by the TargetLynx software were multiplied by a factor of 0.64, which resulted from the ratio of the masses (178.27 g/mL for propofol and 272.40 g/mL for the propofol derivative) multiplied by 0.983 for the purity of the standard.

### 2.9. Method Validation and QC Samples

The method was validated following the guidelines of the Food and Drug Administration (FDA) [14]. Linearity was assessed using six freshly prepared calibration samples in the range of 50–10,000 ng/mL. The calibration curve was determined with TargetLynx software Version 4.1 (Waters) using the least squares method with 1/*x*^2^ weighting. To assess the accuracy and precision of the method, five samples of four different concentrations (50, 100, 1000, and 10,000 ng/mL) were measured on three consecutive days. Both interassay (across all validation sample measurements) and intraassay (within a single day) accuracy and precision were calculated. Precision was expressed as relative standard deviation (RSD): %RSD = 100 ∗ (SD/mean). Accuracy was expressed as the relative deviation of the measured concentration (*C*_measured_) from the nominal concentration (*C*_nominal_): % RE = 100 ∗ (*C*_measured_ − *C*_nominal_)/*C*_nominal_. Precision and accuracy were considered acceptable when %RSD was less than 15 and when % RE was within ±15. During the experiments, additional QC samples were measured at 100, 1000, and 7500 ng/mL, respectively.

### 2.10. Assessment of Long-Term Stability

Using the concentrations measured in 2015 (*C*_2015_) and in 2023 (*C*_2023_), the long-term stability of the propofol plasma concentrations was quantified by the concentration difference (*C*_2023_ − *C*_2015_) and by the relative deviation % RE = 100 ∗ (*C*_2023_ − *C*_2015_)/*C*_2015_ of each sample. Summary statistics were calculated for each patient and for the complete population. Linear regression analyses of *C*_2023_ vs. *C*_2015_ and of % RE vs. *C*_2015_ were further performed. The concentrations measured in 2015 and 2023 were tested for statistically significant difference using the Wilcoxon signed rank test (Statistica 6.0, StatSoft Inc., Tulsa, OK).

## 3. Results and Discussion

### 3.1. Accuracy and Precision

A linear calibration curve (*r*^2^ = 0.999) was determined for the concentration range of 50–10,000 ng/mL. The lower limit of quantification (LLOQ) and the limit of detection (LOD) were 50 and 0.5 ng/mL, respectively. Inter- and intraassay errors are summarized in Table 1. Precision and accuracy were high with %RSD < 3 and %RE within ±5.

The QC samples measured during the experiments also revealed a high accuracy and precision at 100 ng/mL (%RSD = 4.3, %RE = −3.7, *n* = 5), 1000 ng/mL (%RSD = 7.5, %RE = −2.4, *n* = 3), and 7500 ng/mL (%RSD = 1.5, %RE = −0.86, *n* = 4), respectively.

The measurement method described by Eisenried et al. [12] was successfully adapted and re-validated. The same solutions and reagents were used for the derivatization. In contrast to the originally described method, protein precipitation was carried out with methanol to accelerate sample preparation. Instead of the prescribed 500 μL, only 50 μL of sample plasma was needed due to this modification.

### 3.2. Long-Term Stability

**Figure 1:**
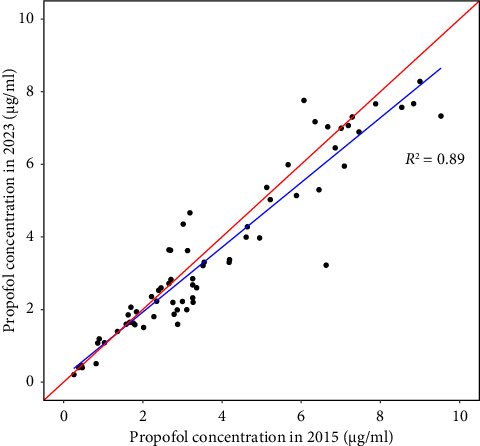
Propofol concentration measurements in 2015 and in 2023. The blue line shows a linear regression through the data, and the red line is the line of identity. *R*^2^: regression coefficient.

The measured propofol concentrations ranged from 0.26 to 9.5 μg/mL in 2015 and from 0.21 to 8.3 μg/mL in 2023, respectively. Linear regression analysis revealed a high correlation between the measurements determined in 2015 and 2023, with the measurements in 2023 being slightly lower than in 2015 (*y* = 0.89 ∗ *x* + 0.14, *r*^2^ = 0.89, *p* < 0.001, Figure 1).

**Figure 2:**
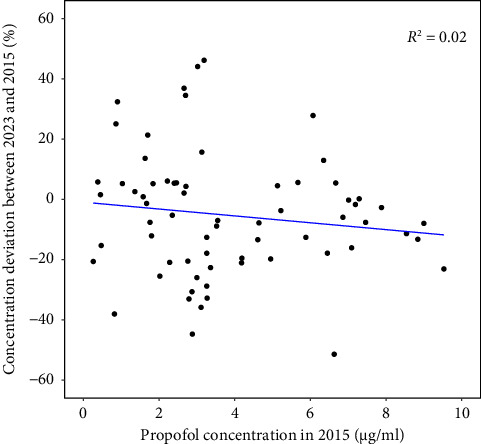
Relative deviation between the propofol plasma concentrations measured in 2023 and in 2015 plotted vs. the propofol plasma concentration measured in 2015. Each point represents one sample. The blue line shows a linear regression through the data. *R*^2^: regression coefficient.

Table 2 presents the propofol plasma concentrations measured in 2023 and 2015. Overall, the median relative deviation between the measurements was −7.0% (interquartile range: −19.7%–5.3%, *p* = 0.0053). No statistically significant correlation was observed between the relative deviation and the propofol plasma concentration (*r*^2^ = 0.02, *p* = 0.28; see Figure 2).

The present study showed that the propofol concentration in blood plasma remained relatively stable when stored at −20°C for eight years. In their study from 2004, Bienert et al. reported that propofol was stable in plasma at −20°C for 60 days but not in whole blood [9]. This study demonstrates that plasma is the optimal matrix for determining propofol concentrations in human specimens and that the drug remains relatively stable over several years.

Propofol is available in various formulations which differ in the use of MCT or long-chain triglyceride (LCT) emulsion. Since propofol with MCT was administered in this study, the present results are only valid for this formulation. However, since the fatty components of the emulsion are subject to lipid metabolism and should not be present in the centrifuged plasma samples, it is unlikely that the specific propofol formulation has an effect on the stability of propofol plasma concentrations.

One limitation of the present study is that it was not designed as a prospective study. Propofol was measured only at the beginning of the eight-year period in 2015 and at the end in 2023. It would certainly have been of scientific interest to continuously determine the change in propofol concentration over this period. However, as the propofol concentration only decreased on average by about 5% over the entire period, detailed analyses with a higher temporal resolution would probably not have provided significant additional information.

Another limitation is that the plasma samples could not be compared using exactly the same method as in 2015. In the meantime, new measurement methods have come onto the market that require less blood or plasma for the measurement than 10 years ago. As less blood is required for the measurements, these methods are also more patient-friendly, which is particularly advantageous for patients with anemia or similar hematological diseases. The following methodological changes were introduced: a revised sample extraction procedure using a reduced sample volume, implementation of a different chromatographic column, and transition to a new LC–MS/MS instrument. Despite these adjustments, the core elements of the method, including analyte chemistry, internal standard composition, and overall analytical concept, remained unchanged. Such modifications fall within the scope of partial validation, which is appropriate for changes in sample preparation, chromatographic conditions, or instrumentation. The new measurement method was validated before use, and its accuracy and precision were determined. These assessments demonstrated that the modified method meets the performance criteria and remains suitable for the intended use. The gold standard would be to use exactly the same method. However, when the measurement interval exceeds more than 5 years, this is hardly feasible due to technical advances.

## 4. Conclusion

In conclusion, this analysis provides convincing results for a relatively good agreement between the measurements in 2015 and 2023. This suggests that the concentration of propofol in human plasma remains relatively stable under the standard storage conditions even for eight years.

## Figures and Tables

**Table 1:** Inter- and intraassay errors of the applied analytical method.

	Nominal concentration (ng/mL)	Mean ± SD (ng/mL)	% RSD	% RE	*n*
Interassay	50	50.1 ± 1.4	2.7	0.11	15
	100	103 ± 2.4	2.3	2.8	15
	1000	1045 ± 25	2.4	4.5	15
	10,000	10,157 ± 260	2.6	1.6	15

Intraassay	50	50.7 ± 1.1	2.2	1.5	5
	100	104 ± 2.0	1.9	3.7	5
	1000	1035 ± 17	1.6	3.5	5
	10,000	10,115 ± 264	2.6	1.2	5

*Note:* % RSD, relative standard deviation; %RE, relative error from nominal concentration.

Abbreviation: SD, standard deviation.

**Table 2:** Propofol plasma concentrations measured in 2023 and in 2015.

Sample	Patient #1			Patient #2			Patient #3			Patient #4			Patient #5		
	*C*_2015_ (μg/mL)	*C*_2023_ (μg/mL)	% RE	*C*_2015_ (μg/mL)	*C*_2023_ (μg/mL)	% RE	*C*_2015_ (μg/mL)	*C*_2023_ (μg/mL)	% RE	*C*_2015_ (μg/mL)	*C*_2023_ (μg/mL)	% RE	*C*_2015_ (μg/mL)	*C*_2023_ (μg/mL)	% RE
1	8.54	7.57	−11.4	7.01	6.99	−0.2	4.95	3.97	−20	7.29	7.30	0.2	6.07	7.76	27.8
2	9.00	8.28	−8.0	6.35	7.17	13.0	2.76	2.19	−21	1.58	1.59	0.9	2.28	1.80	−20.9
3	8.84	7.67	−13.2	7.19	7.07	−1.7	3.27	2.20	−33	2.79	1.87	−33.1	3.26	2.32	−28.8
4	1.80	1.58	−12.1	2.66	2.72	2.1	4.18	3.30	−21	2.71	2.83	4.3	3.11	2.00	−35.9
5	3.26	2.85	−12.6	1.36	1.40	2.6	4.19	3.37	−20	7.09	5.95	−16.1	9.53	7.33	−23.1
6	3.55	3.30	−7.0	2.40	2.53	5.4	4.61	3.99	−13	5.88	5.14	−12.6	6.63	3.22	−51.4
7	3.52	3.21	−8.9	2.46	2.60	5.5	6.45	5.30	−18	4.64	4.28	−7.8	6.86	6.45	−5.9
8	2.66	3.64	36.9	6.67	7.03	5.4	3.26	2.68	−18	7.46	6.89	−7.6	3.13	3.62	15.7
9	3.19	4.66	46.2	5.67	5.99	5.6	3.36	2.60	−23	7.88	7.67	−2.7	2.35	2.23	−5.3
10	3.02	4.35	44.1	5.13	5.36	4.5	2.87	1.99	−31	1.70	2.06	21.4	3.00	2.22	−26.0
11	2.70	3.63	34.5	5.22	5.03	−3.7	2.88	1.59	−45	0.38	0.40	5.8	2.02	1.51	−25.5
12	1.67	1.65	−1.4	2.22	2.36	6.1	0.82	0.51	−38				0.47	0.40	−15.3
13	1.76	1.63	−7.6	1.84	1.94	5.2									
14	0.90	1.19	32.4	1.63	1.85	13.6									
15	0.86	1.08	25.1	1.03	1.08	5.2									
16	0.26	0.21	−20.6	0.45	0.46	1.6									

Median			−7.3			5.2			−20.8			−2.7			−22.0
Q25			−11.6			2.0			−31.2			−0.2			−26.7
Q75			32.9			5.5			−9.1			2.6			−5.8

*Note:* %RE, relative deviation; Q25, 25% quantile; Q75, 75% quantile.

## Data Availability

The data that support the findings of this study are available from the corresponding author upon reasonable request.
